# Multidirectional Effects of Acerola and Ginger Extracts on the Quality of Dry-Cured Ham

**DOI:** 10.3390/foods14244207

**Published:** 2025-12-07

**Authors:** Robert Pietrasik, Sylwester Pawęta, Grażyna Budryn, Joanna Grzelczyk, Zuzanna Paprocka, Małgorzata Zakłos-Szyda

**Affiliations:** 1 Institute of Materials Science and Engineering, Faculty of Mechanical Engineering, Lodz University of Technology, Stefanowskiego 1-15, 90-924 Lodz, Poland; robert.pietrasik@p.lodz.pl (R.P.); sylwester.paweta@p.lodz.pl (S.P.); 2 Quality Meat Pawęta Ltd., Kolonia 2a, Krokocice, 98-240 Szadek, Poland; 3 Institute of Food Technology and Analysis, Faculty of Biotechnology and Food Sciences, Lodz University of Technology, Stefanowskiego 2-22, 90-537 Lodz, Poland; grazyna.budryn@p.lodz.pl (G.B.); zuzanna.paprocka@p.lodz.pl (Z.P.); 4 Institute of Molecular and Industrial Biotechnology, Faculty of Biotechnology and Food Sciences, Lodz University of Technology, Stefanowskiego 2-22, 90-537 Lodz, Poland; malgorzata.zaklos-szyda@p.lodz.pl

**Keywords:** pork, dry-curing, acerola, ginger, microbial starters, *Lactobacillus sakei*, *Pediococcus acidilactici*, *Penicillium nalgiovense*

## Abstract

Meat, as a primary source of complete protein, vitamins, and minerals, remains a key component of the human diet despite controversies surrounding its production. The aim of this study was to develop a production technology for dry-cured ham with reduced nitrite content, using natural plant extracts (from acerola and ginger) and microbial (*Lactobacillus sakei*, *Pediococcus acidilactici*) and mold (*Penicillium nalgiovense*) starter cultures. The process included tumbling, fermentation, and curing under various conditions, including vacuum. Physicochemical analyses (pH, shear force, color, WHC, TBARS, salt and nitrites content) and sensory evaluation were conducted. Results showed that the synergistic use of plant extracts and microbial additives enabled the production of high-quality ham with desirable sensory properties, reduced lipid oxidation when vacuum curing or additional stocking was applied (TBARS < 2 mg MDA/kg), stable color, and optimal pH (5.6–5.7). Vacuum curing reduced weight loss, while smoking improved sensory traits. The proposed technology offers a promising alternative for industrial meat production, allowing for reduced chemical additives while maintaining safety, quality, and consumer acceptability.

## 1. Introduction

Meat is a major source of highly digestible protein, providing all essential amino acids in proper proportions. It also supplies vitamins and minerals, some with much higher bioavailability in animal products, such as iron—critical for pregnant women [1,2]. Despite environmental harm, ethical concerns, certain health risks (e.g., carcinogenic and dyslipidemic effects), and high production costs, societies continue consuming meat due to its proven health benefits. Studies linking affluence to higher meat intake confirm strong demand. Alongside developing meat substitutes, improving processing methods to ensure maximum consumer safety remains essential [3].

One method to enhance meat safety is dry curing. This practice is deeply rooted in Mediterranean culinary traditions (Italy, Spain, France), where the climate favors production and shapes a flavor and aroma profile highly valued by consumers [4,5]. In Central Europe, curing requires specialized chambers to control temperature and humidity [6]. The process uses chemical additives, with nitrite salt being crucial; its content in dry-cured hams meets new regulations [7]. Nitrite is applied for its antimicrobial effect, preventing *Clostridium botulinum* growth and toxin formation [8]. In anaerobic, acidic conditions, nitrite converts to nitric oxide, which binds to myoglobin, forming nitrosomyoglobin and giving meat its bright red color [9]. It also contributes to aroma and texture by reacting with muscle proteins (myosin, actin), maintaining a gelled structure and inhibiting lipid oxidation [10]. Nitrite impacts human health as a source of endogenous nitric oxide, regulating blood pressure, wound healing, immunity, and neurological processes, and may help prevent cardiovascular diseases. However, high nitrite levels can form carcinogenic nitrosamines in the stomach’s acidic environment [11].

Meat producers face the challenge of finding alternatives that replicate nitrite’s technological functions while reducing its use in line with new regulations [7]. Strategies include natural nitrate sources such as nitrate-rich plants (broccoli, celery, spinach, lettuce, parsley, beetroot, chard, radish), plant-based colorants (phenolic compounds from raspberry, tea, red grapes, blackcurrant), microbial fermentation products (pigment from *Monascus purpureus*), animal-derived dyes (carmine from cochineal insects), and new processing technologies combining various additives [12,13]. These approaches are crucial for dry curing without thermal processing, where toxic mold development remains a major concern, making mold control and mycotoxin reduction essential [14].

Ginger is a promising additive due to gingerols, acting as a natural preservative and antioxidant. It inhibits lipid and protein oxidation, improves durability and stability, and its proteolytic activity tenderizes muscles by breaking down fibers and connective tissue, enhancing water-holding capacity and juiciness. Ginger may also suppress certain toxic microorganisms [15]. Acerola addition improves lipid stability and significantly enhances cured meat color. Its high ascorbic acid content accelerates curing by promoting nitrite conversion to nitric oxide. However, acerola’s acidity may require adjusting its amount to maintain optimal pH for protease activity, and the appropriate levels of acerola and ginger need sensory evaluation [16].

Dry-cured meats, despite low water activity, support complex mycobiota such as molds from *Penicillium* and *Aspergillus*, adapted to xerophilic and halophilic conditions [17]. While most molds are non-toxic, strains producing mycotoxins, including *P. nordicum*, *P. verrucosum*, and *A. westerdijkiae*, have been isolated. Using microbiological starters effectively limits undesirable mold contamination in both industrial and traditional products. Functional starters must lack mycotoxin and antibiotic production and compete well in the environment; examples include *P. nalgiovense* and *P. chrysogenum* [18,19]. Acidifying bacteria accelerate pH reduction and muscle proteolysis; *Lactobacillus sakei* offers short fermentation, Mediterranean flavor, and rapid pH drop. Recently, *Pediococcus acidilactici* has been recognized as an additional acidifying culture improving safety [20]. Starter cultures also reduce aflatoxin production, and mixed-strain starters for controlling toxic substances in dry-fermented meats are considered a promising biological control approach [21].

Not all types of climate allow curing without specialized chambers controlling temperature and humidity. Although such chambers require dedicated installations, they enable unusual conditions like specific atmospheres and pressure. This study was the first to apply vacuum curing of ham combined with other technological modifications. The goal was to produce dry-cured ham with low nitrite content, compliant with new regulations, using natural plant extracts and microbial starters while maintaining high efficiency and quality. The study evaluated the effects of acerola and ginger extracts combined with factors such as sugar addition, smoking, vacuum curing, and starter activity on physicochemical and sensory properties of pork ham. The hypothesis was that the simultaneous use of plant-derived additives and microbiological starters under selected conditions provides better ham quality than the use of microbiological cultures alone.

## 2. Materials and Methods

### 2.1. Raw Materials, Chemicals and Reagents

The pork *biceps femoris* muscle was supplied by local slaughterhouse. It was stored for 24 h post-slaughter at 4 °C. In this study, the *biceps femoris* muscle was used alone due to methodological and technological reasons. This reduced sample variability and ensured reproducibility. Its size, shape, and structure facilitated uniform curing and drying. Acerola extract was provided by Biomus, Ltd. (Lublin, Poland), and ginger extract by Guangzhou Dugai Trading Co., Ltd. (Guangzhou, China). Non-iodized sea salt came from Italmex, Ltd. (Macierzysz, Poland), and curing salt from Planteon, Ltd. (Żelazków, Poland). Microbial starters *L. sakei* subsp. *Sakei* WDCM 00015 ATCC^®^ 15521 and *P. acidilactici* ATCC^®^ 8042 were obtained from Polaura, Ltd. (Łódź, Poland), and *P. nalgiovense* LYOCARNI FPN-07 from Swojski Wyrób, Ltd. (Polanka Wielka, Poland). Filter paper, 1,1,3,3-tetramethoxypropane, 0.1 mol/L HCl, 2-thiobarbituric acid (TBA), butylhydroxytoluene (BHT), silver nitrate, sodium vernate (EDTA), 5% trichloroacetic acid (TCA) solution, acidic sulfanilamide and N-(1-naphthyl)-ethylenediamine dichloride were supplied by Polaura, Ltd. (Łódź, Poland).

### 2.2. Technological Process

The experimental design included 21 pork ham variants, each representing a unique combination of factors (Table 1). Tenderizing was carried out at 3 °C for 3 days at 80% relative humidity. All samples were prepared with a reduced nitrite content of 80 mg/kg of fresh meat. The control sample (variant 1a) contained the same nitrite level, as well as salt, glucose and *L. sakei*, present in all samples, without the addition of plant extracts, and served as a reference point for assessing the effects of the other variables.

The study initially assumed a complex experimental design that considered combinations of several technological factors and functional additives. The goal was to investigate their interactions and synergistic effects on the quality of cured ham. In a full factorial design, the number of variants depends on the number of variables and their levels. The final research design would have included over 300 variants. In this situation, a fractional factorial design (1/16) was used to effectively examine the effects of selected variables with a limited number of variants, reducing the number of experiments to 21 while retaining information on the main effects and key interactions. In this way, a balance was maintained between the effectiveness of the study and its comprehensiveness while maintaining the statistical power of the analysis. The variants were selected based on previous studies taking into account the combinations with the greatest potential to influence the product quality and the possibility of identifying key factors influencing the sensory quality, lipid stability and texture of the hams.

Ultimately, the used additives included: sea salt—fixed amount (10 g/kg), mineral salt—fixed amount (20 g/kg including nitrate and nitrite), glucose—variable (1, 6, or 12 g/kg), *L. sakei*—variable (0.2–0.25 g/kg), *P. acidilactici*—presence/absence (0.25 g/kg), acerola powder—variable (0, 0.3, 0.5 or 2 g/kg), ginger—presence/absence (1 g/kg), *P. nalgiovense*—presence/absence (0.5 g/kg).

Tumbling was performed in cycles of 50 min of tumbling and 10 min of rest for 4 h. Aging was then carried out in a cold room at 3 to 8 °C, under atmospheric or reduced pressure (800 or 900 mBa) for 6 to 10 days depending on the variant. The meats were then netted and hung. In some variants meats were packed in an additional casing or vacuum-cured in a special chamber at 120 mBa. The curing and fermentation process was conducted at 17 to 25 °C for 9 to 12 days, depending on the variant. Smoking (3 h, 20 °C) was applied to some variants. The meat was then stabilized at 14 °C for 6 days and subjected to analysis. The ham processing was carried out using the following equipment: vacuum massaging/tumbling—Servotech type 600/800 (Rzeszów, Poland), cold smoking—JMJ Worońko semi-automatic chamber (Wrocław, Poland), curing chamber of our own design and construction based on the ClimaGold OPAL-N-2-P-CHf/He—FW-We-630 ventilation unit and ActivePure RCI air purification technology. Minimum filtration class compliant with ISO 16890: ePM10 75% and ePM1 55%, and microbiological purity in terms of reducing the following pathogens: mold spores—minimum 99.5% reduction in a 24 h test, *E. coli*—minimum 99.5% reduction in a 15 min test, *Staphylococcus aureus*—minimum 99.5% reduction in a 30 min test, *Legionella*—minimum 99.5% reduction in a 30 min test, *Norovirus*—minimum 93% reduction in a 30 min test, *Clostridium difficile*—minimum 85% reduction in a 30 min test.

The 21 variants for producing cured ham are presented below in coded form to facilitate the identification of conditions in variants 1 to 6. The codes use the following abbreviations: variant number, e.g., 1a: temperature and days of aging, and optional vacuum applied to aging, e.g., 7/6V_glucose addition, e.g., G1_acerola and ginger extract addition, e.g., 0.3/1_microbial starters *L. sakei*, *P. acidilactici*, *P. nalgiovense* addition, e.g., 0.2/0/0_temperature and curing time, and optional vacuum applied to curing, e.g., 25/9V_use of stocking A, use of smoking S. The identifying codes are listed below:

1a: 3/10__G6___0/0___0.2/0/0______17/12_S
1b: 3/10__G6___0/0___0.2/0/0.5____17/12
2a: 3/8___G6___0.3/1__0.2/0/0_____21/11
2b: 3/8___G6___0.3/1__0.2/0/0_____21/11_S
2c: 3/8___G6___0.3/1__0.2/0/0.5____21/11
2d: 3/8___G12__0.3/1__0.2/0/0_____21/11
2e: 3/8___G12__0.3/1__0.2/0/0_____21/11_S
2f: 3/8___G12__0.3/1__0.2/0/0.5____21/11
3a: 7/6V__G1__0.5/1__0.2/0.25/0___25/9
3b: 7/6V__G1__0.5/1__0.2/0.25/0___25/9_A
3c: 7/6V__G1__0.5/1__0.2/0.25/0.5__25/9
3d: 7/6V__G1__0.5/1__0.2/0.25/0.5_25/9_A
4a: 3/6V__G1__2/1____0.25/0/0____25/9
4b: 3/6V__G1__2/1____0.25/0/0____25/9_S
4c: 3/6V__G1__2/1____0.25/0/0____25/9V
4d: 3/6V__G1__2/1____0.25/0/0____25/9V_S
5a: 3/8____G6__2/1____0.25/0/0____25/9
5b: 3/8____G6__2/1____0.25/0/0____25/9_A
5c: 3/8____G6__2/1____0.25/0/0____25/9_S
5d: 3/8____G6__2/1____0.25/0/0____25/9_SA
6: 8/10___G12_2/1____0.25/0/0____25/9

### 2.3. Physicochemical Analyses

#### 2.3.1. pH Measurement

After stabilization, 4 g of the sample and 16 mL of distilled water were mixed and homogenized at 20 °C for 1 min. The homogenate was then measured using a pH meter with a glass electrode (model S220, Mettler-Toledo, Schwerzenbach, Switzerland) [22].

#### 2.3.2. Shear Force Measurement

The test was performed using a Zwick 1120 texture analyzer (Zwick GmbH&Co., Ulm, Germany) equipped with a Warner-Bratzler flat blade attachment. The maximum force required (Zwick GmbH&Co., Ulm, Germany) to cut through a product sample (1 cm thick and 2 cm wide slice) was measured. The result was expressed in N. The crosshead speed during the test was 50 mm/min. Sample temperature during testing: 6–8 °C. Twelve measurements (cuts) were performed for each ham [23].

#### 2.3.3. Color L*a*b* Parameters

Color parameters L*, a*, b* were measured using a Minolta CR-400 colorimeter (Ramsey, NJ, USA) on the cross-section, immediately after slicing the product [24].

#### 2.3.4. Water-Holding Capacity (WHC)

The meat was ground using a meat grinder with a 3 mm plate. Portions of 300 mg of meat were taken for WHC determination. WHC is the ability of meat to retain free water despite the application of force (load) to facilitate the release of muscle juice. WHC was determined using the filter paper method. This method involved using filter paper to absorb meat juice released from the sample under mechanical pressure. Hard filter paper, previously dried to constant weight and conditioned in a desiccator, was used. To determine the water absorption capacity of the filter paper, 0.3 mL of distilled water was applied to it. The amount of water (mL) per 1 cm^2^ of stain was determined. Weighed meat samples of 300 mg were placed in the center of the paper and loaded with a 1 kg weight for 20 min. After this time, the area of the meat juice stain and the meat stain were measured. The difference in measured areas was used to calculate the water-holding capacity of the meat, which was related to the mass of the tested sample and expressed as a percentage. The higher the percentage, the more water is released from the meat, indicating lower WHC [25].

#### 2.3.5. Lipid Oxidation Index (TBARS)

The degree of lipid oxidation was assessed as substances reacting with TBA (TBARS). To calibrate the reaction TBA reacted with malondialdehyde (MDA), resulting in the formation of a colored compound. The TBARS value was expressed as mg MDA/kg of meat. MDA for the standard curve was obtained by acid hydrolysis of a 1,1,3,3-tetramethoxypropane solution in 0.1 M HCl in a water bath for 5 min. MDA solutions were prepared for the calibration curve with concentrations of 0.1748, 0.1398, 0.1049, 0.0699, and 0.0346 μg/mL. For TBARS determination, 5 g of minced meat sample was weighed, 0.5 mL of EDTA was added and mixed, followed by 2.5 mL of 0.8% BHT, mixed again, and then 4 mL of 5% TCA was added and homogenized in a vortex at 2000 rpm for 5 min. The sample was filtered using filter paper. The filtered sample was transferred to a 10 mL volumetric flask and filled up with 10% TCA. From the flask, 4 mL was transferred to a test tube, and 1 mL of TBA was added and sealed with aluminum foil. A blank sample was prepared in a separate tube using 0.5 mL of EDTA, 2.5 mL of 0.8% BHT, 4 mL of 5% TCA and 1 mL of TBA. The tubes were incubated in a water bath at 95 °C for 90 min. After this time, the tubes were cooled, and absorbance was measured against the blank sample using a UV-VIS spectrophotometer (Shimadzu 2000A) at a wavelength of 532 nm. Results were quantified as malondialdehyde equivalents (sample mg/MDA/kg) [26].

#### 2.3.6. Salt Content

Natrium chloride concentration was calculated from chloride titration. Chloride anions were titrated in ashed (by furnace) samples with silver nitrate using the Mohr method [27].

#### 2.3.7. Weight Loss

Weight loss was calculated based on the initial weight of the meat plus the weight of additives and the weight after stabilization [28].

### 2.4. Nitrate and Nitrite Content

Determination of nitrate and nitrite content in meat products was carried out after extracting from the sample, followed by deproteinization and filtration. The filtrate containing nitrates and nitrites was then passed through a cadmium column resulting in reducing nitrates to nitrites, and after adding an acidic sulfanilamide solution, the nitrites created a diazonium compound coupled with N-(1-naphthyl)-ethylenediamine dichloride, forming a purple azo dye measured at a wavelength of 540 nm. The nitrites were measured without passing the column [29].

### 2.5. Sensory Evaluation

Fifteen sensory panelists evaluated the sensory properties of dry-cured ham samples using a basic taste identification test. The panelists were academic staff from the Faculty of Biotechnology and Food Sciences and students who were trained for 7 h using commercially available ham products. The sample was sliced into a thickness of 0.2 mm and presented. The quantitative descriptive analysis (QDA) was applied. The sensory evaluation included texture, tenderness, off-flavor, flavor, saltiness and sourness rated on a 10-point scale: 10 = exceptionally good or desirable, 1 = extremely bad or undesirable [30]. The overall acceptability was than calculated as the mean of the individual features [31]. Each panelist received 3 coded samples per session. Each sample was served randomly with a 3 min interval between evaluations.

### 2.6. Statistical Analysis

The study employed 21 technological variants, each analyzed in six independent replicates. The study was designed as a completely randomized experimental design (CRD). Each replicate used different muscle, for a total of 126 meat samples from about 65 animals. All samples were randomly assigned to variants, and processing conditions were controlled and reproducible. This allowed us to focus on the effects resulting from the technological variables used. Main effects referred to the type of additive (glucose, acerola, ginger), the presence of starters (*L. sakei*, *P. acidilactici*, *P. nalgiovense*), and curing conditions, while random effects referred to individual differences between muscles from different animals, which were treated as a random biological factor. Three parallel physicochemical and sensory analyses were performed for each of the 126 samples, which were not treated as separate replicates but as a composite characterization of a single sample.

The physicochemical properties were analyzed using one-way analysis of variance and correlation analysis. Values are expressed as means ± SD, and significant differences between mean values were determined by Duncan’s multiple range test (*p* < 0.05). Correlation was calculated as Pearson’s correlation coefficient. Statistica 10.0 was used for statistical analysis.

## 3. Results and Discussion

### 3.1. Weight Loss

**Figure 1:**
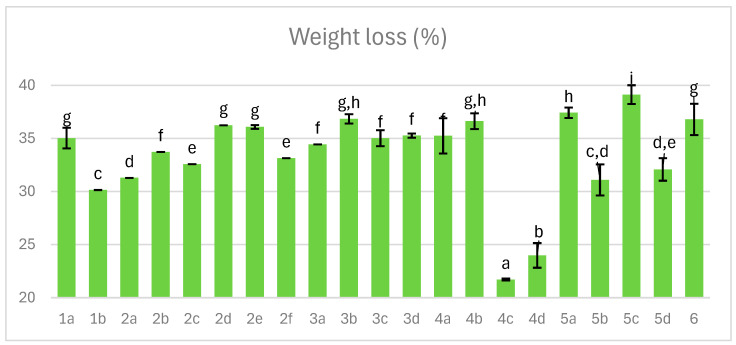
The effect of production technology variants and additives on weight loss of dry-cured pork hams. Variants labels (for details see Table 1): 1a: 3/10__G6___0/0___0.2/0/0______17/12_S; 1b: 3/10__G6___0/0___0.2/0/0.5____17/12; 2a: 3/8___G6___0.3/1__0.2/0/0_____21/11; 2b: 3/8___G6___0.3/1__0.2/0/0_____21/11_S; 2c: 3/8___G6___0.3/1__0.2/0/0.5____21/11; 2d: 3/8___G12__0.3/1__0.2/0/0_____21/11; 2e: 3/8___G12__0.3/1__0.2/0/0_____21/11_S; 2f: 3/8___G12__0.3/1__0.2/0/0.5____21/11; 3a: 7/6V__G1__0.5/1__0.2/0.25/0___25/9; 3b: 7/6V__G1__0.5/1__0.2/0.25/0___25/9_A; 3c: 7/6V__G1__0.5/1__0.2/0.25/0.5__25/9; 3d: 7/6V__G1__0.5/1__0.2/0.25/0.5_25/9_A; 4a: 3/6V__G1__2/1____0.25/0/0____25/9; 4b: 3/6V__G1__2/1____0.25/0/0____25/9_S; 4c: 3/6V__G1__2/1____0.25/0/0____25/9V; 4d: 3/6V__G1__2/1____0.25/0/0____25/9V_S; 5a: 3/8____G6__2/1____0.25/0/0____25/9; 5b: 3/8____G6__2/1____0.25/0/0____25/9_A; 5c: 3/8____G6__2/1____0.25/0/0____25/9_S; 5d: 3/8____G6__2/1____0.25/0/0____25/9_SA; 6: 8/10___G12_2/1____0.25/0/0____25/9. Different letters above the bars indicate statistically significant differences between variants; n = 6.

From the producer’s perspective, the critical parameters defining dry-cured ham encompass both superior quality and process profitability, which are directly associated with yield, i.e., the final product weight. The predominant cause of weight reduction is water loss, strongly influenced by the duration of processing steps such as tumbling, aging, curing, optional smoking, and stabilization. Figure 1 illustrates weight loss across variants of dry-cured ham. The most favorable outcomes were achieved under vacuum curing at 120 mBa, where weight loss was limited to 22–24%, depending on whether cold smoking for 3 h was additionally applied (variants 4c,d). In other cases, weight loss ranged from 30% to 39%. Notably, mold inoculation prior to curing consistently mitigated weight loss, as observed in comparisons of variants 1b vs. 1a, 2f vs. 2d, and 3d vs. 3b.

Inoculation with molds, particularly species of the genus Penicillium, during curing appears to reduce final weight loss through complex physicochemical and microbiological mechanisms affecting water migration and surface protection. One such mechanism involves the formation of a physical barrier: surface colonization by mold results in a dense mycelial layer acting as a semi-permeable membrane that restricts water evaporation from the meat interior.

Additionally, mold enzymatic activity may locally increase concentrations of water-binding functional groups (carboxyl, hydroxyl, amino), thereby reducing surface water activity and lowering the vapor pressure gradient between the chamber and the ham interior, ultimately limiting evaporation [32,33]. *P. nalgiovense*, forming a protective biofilm and producing antifungal proteins, may further inhibit the growth of mycotoxin producing molds [34].

As demonstrated by Kim et al. (2023), curing at 25 °C, compared to 20 °C, promotes robust growth of *P. nalgiovense* and may contribute to reduced water loss, a trend confirmed in our study except for variants involving reduced-pressure curing or additional stocking [19]. Smoking, conversely, induces protein denaturation on the ham surface, forming a crust that could retain moisture during final stabilization; however, this effect was not confirmed under the low smoking temperatures applied (variants: 2b vs. 2a, 2e vs. 2d, 4b vs. 4a, 4d vs. 4b, 5c vs. 5a, and 5d vs. 5b) [35,36,37].

Proteolysis, previously highlighted as a key determinant of raw meat quality, water retention, and juiciness, is positively influenced by inoculation with lactic acid bacteria starters. In this study, *L. sakei* was applied in all variants, combined with *P. acidilactici* in variants 3a–d. A commercial strain of *L. sakei* was inoculated with glucose as a carbon source [38]. Beyond water retention, inoculation exerts additional effects. Martín et al. (2021) demonstrated that such starters reduce *Enterobacteriaceae* counts to undetectable levels and *Listeria monocytogenes* to approximately 2 log CFU/g by the end of processing [39]. Moreover, dry-cured products inoculated with *L. sakei* and *P. acidilactici* exhibit angiotensin-converting enzyme (ACE) inhibitory activity and antioxidant properties, attributed to bioactive peptide formation involved in antihypertensive processes and metabolic regulation. These bacteria thus play a pivotal role in conferring functional health benefits to dry-cured hams, a feature that will be addressed in a separate publication [40].

### 3.2. pH Changes

**Figure 2:**
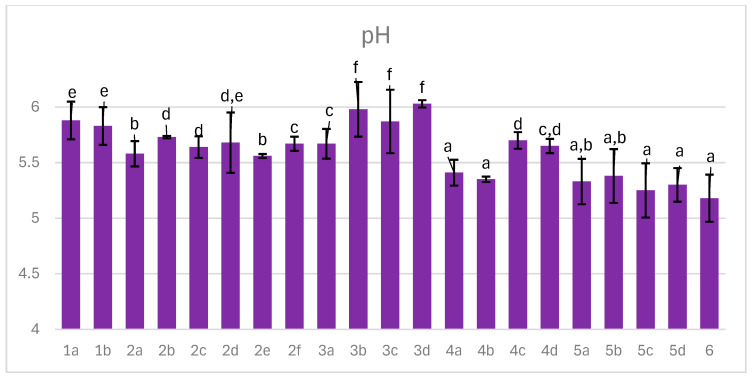
The effect of production technology variants and additives on pH value of dry-cured pork hams. Variants labels (for details see Table 1): 1a: 3/10__G6___0/0___0.2/0/0______17/12_S; 1b: 3/10__G6___0/0___0.2/0/0.5____17/12; 2a: 3/8___G6___0.3/1__0.2/0/0_____21/11; 2b: 3/8___G6___0.3/1__0.2/0/0_____21/11_S; 2c: 3/8___G6___0.3/1__0.2/0/0.5____21/11; 2d: 3/8___G12__0.3/1__0.2/0/0_____21/11; 2e: 3/8___G12__0.3/1__0.2/0/0_____21/11_S; 2f: 3/8___G12__0.3/1__0.2/0/0.5____21/11; 3a: 7/6V__G1__0.5/1__0.2/0.25/0___25/9; 3b: 7/6V__G1__0.5/1__0.2/0.25/0___25/9_A; 3c: 7/6V__G1__0.5/1__0.2/0.25/0.5__25/9; 3d: 7/6V__G1__0.5/1__0.2/0.25/0.5_25/9_A; 4a: 3/6V__G1__2/1____0.25/0/0____25/9; 4b: 3/6V__G1__2/1____0.25/0/0____25/9_S; 4c: 3/6V__G1__2/1____0.25/0/0____25/9V; 4d: 3/6V__G1__2/1____0.25/0/0____25/9V_S; 5a: 3/8____G6__2/1____0.25/0/0____25/9; 5b: 3/8____G6__2/1____0.25/0/0____25/9_A; 5c: 3/8____G6__2/1____0.25/0/0____25/9_S; 5d: 3/8____G6__2/1____0.25/0/0____25/9_SA; 6: 8/10___G12_2/1____0.25/0/0____25/9; Different letters above the bars indicate statistically significant differences between variants; n = 6.

The pH of dry-cured ham is a critical determinant of texture, microbial safety, and biochemical stability, with an optimal range of 5.6–6.2 that supports proteolysis while inhibiting spoilage organisms. Values below this range may cause excessive acidity and undesirable texture, whereas levels above 6.2 increase the risk of microbial contamination and formation of harmful metabolites. Figure 2 illustrates pH values detected in hams processed under different technological variants.

Measured pH ranged from 5.18 to 6.03, remaining within the safety threshold. A pH below 6.0 generally correlates with the desired texture of dry-cured ham. Under these conditions, the gel formed by coagulated solubilized proteins stabilizes structural cohesion, while reduced water retention facilitates efficient drying [41]. The 80% humidity maintained in the curing chamber promoted protein hydrolysis, favoring endogenous protease activity and resulting in pH reduction close to 5 in several variants, including 4a,b and 5a–d, characterized by high acerola addition rich in organic acids. Low pH can also be attributed to lactic acid production; however, the second bacterial starter did not correlate with further pH decrease (variants 3a–d), likely due to limited glucose supplementation. Although low pH enhances shelf life, values below 5.6 may compromise sensory attributes. Addition of acerola extract up to 0.5% appears to support sensory quality, while vacuum curing achieved an optimal pH of 5.6–5.7 (variants 4c,d), likely due to reduced mass loss and limited oxidative reactions generating acidic metabolites from protein and lipid degradation [42]. Increasing process temperature from 20 °C to 25 °C combined with higher glucose supplementation further decreased pH (variants 5a–d, 6). The optimal growth range for lactic acid bacteria is 25–45 °C; thus, the lower pH observed may result from enhanced bacterial activity during aging at 25 °C, as well as intensified protein degradation linked to increased decarboxylase activity. Smoking can theoretically reduce pH through penetration of acidic volatiles; however, this effect was not confirmed in final products (variants 1a, 2b,e, 4b,d, 5c,d) [43]. The lack of significant impact likely reflects the short duration and low temperature of smoking, limiting volatile absorption [35].

### 3.3. Salt Content

Sodium chloride remains a fundamental component in meat curing, driving essential biochemical reactions and contributing to flavor, texture, and protein functionality. Its technological and microbiological roles make complete elimination impractical, despite health concerns associated with hypertension. This poses a challenge for the meat industry, as health-conscious consumers increasingly restrict intake of dry-cured products. Strategies to reduce sodium include replacing traditional curing salt with sea salt and lowering levels below 3% when supported by additional preservation methods. Nevertheless, sodium chloride is indispensable for inhibiting proteases and maintaining structural integrity, which limits the extent of reduction [36,44,45].

**Figure 3:**
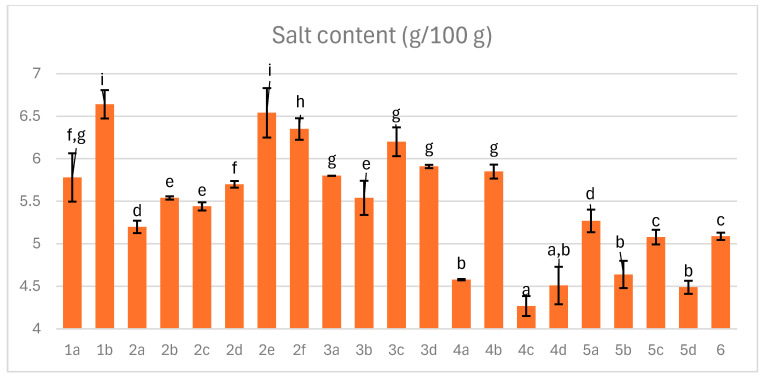
The effect of production technology variants and additives on salt content in dry-cured pork hams. Variants labels (for details see Table 1): 1a: 3/10__G6___0/0___0.2/0/0______17/12_S; 1b: 3/10__G6___0/0___0.2/0/0.5____17/12; 2a: 3/8___G6___0.3/1__0.2/0/0_____21/11; 2b: 3/8___G6___0.3/1__0.2/0/0_____21/11_S; 2c: 3/8___G6___0.3/1__0.2/0/0.5____21/11; 2d: 3/8___G12__0.3/1__0.2/0/0_____21/11; 2e: 3/8___G12__0.3/1__0.2/0/0_____21/11_S; 2f: 3/8___G12__0.3/1__0.2/0/0.5____21/11; 3a: 7/6V__G1__0.5/1__0.2/0.25/0___25/9; 3b: 7/6V__G1__0.5/1__0.2/0.25/0___25/9_A; 3c: 7/6V__G1__0.5/1__0.2/0.25/0.5__25/9; 3d: 7/6V__G1__0.5/1__0.2/0.25/0.5_25/9_A; 4a: 3/6V__G1__2/1____0.25/0/0____25/9; 4b: 3/6V__G1__2/1____0.25/0/0____25/9_S; 4c: 3/6V__G1__2/1____0.25/0/0____25/9V; 4d: 3/6V__G1__2/1____0.25/0/0____25/9V_S; 5a: 3/8____G6__2/1____0.25/0/0____25/9; 5b: 3/8____G6__2/1____0.25/0/0____25/9_A; 5c: 3/8____G6__2/1____0.25/0/0____25/9_S; 5d: 3/8____G6__2/1____0.25/0/0____25/9_SA; 6: 8/10___G12_2/1____0.25/0/0____25/9; Different letters above the bars indicate statistically significant differences between variants; n = 6.

During chamber curing, salt was applied at 3%, achieving approximately 80% absorption efficiency. As shown in Figure 3, sodium chloride content in the final product ranged from 4.3 to 6.6 g/100 g, primarily influenced by mass loss during processing, since initial addition was constant: 10 g/kg of sea salt and 20 g of curing salt (19.92 g mineral salt and 0.08 g nitrites and nitrates mixture).

The correlation coefficient was only 0.35, indicating that mass loss did not proportionally reflect salt concentration. This variation likely resulted from differences in chloride ion binding to proteins, modulated by pH, the degree of protein hydrolysis in part influenced by the chamber temperature [45].

### 3.4. Water-Holding Capacity

**Figure 4:**
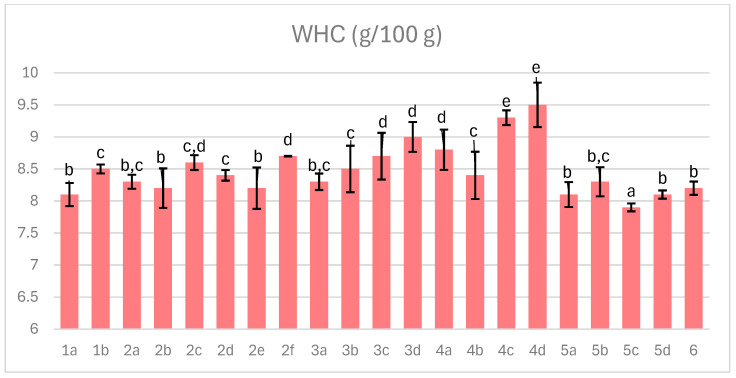
The effect of production technology variants and additives on water-holding capacity of dry-cured pork hams; an increase in WHC indicates, according to the measurement procedure, a greater water release from the meat. Variants labels (for details see Table 1): 1a: 3/10__G6___0/0___0.2/0/0______17/12_S; 1b: 3/10__G6___0/0___0.2/0/0.5____17/12; 2a: 3/8___G6___0.3/1__0.2/0/0_____21/11; 2b: 3/8___G6___0.3/1__0.2/0/0_____21/11_S; 2c: 3/8___G6___0.3/1__0.2/0/0.5____21/11; 2d: 3/8___G12__0.3/1__0.2/0/0_____21/11; 2e: 3/8___G12__0.3/1__0.2/0/0_____21/11_S; 2f: 3/8___G12__0.3/1__0.2/0/0.5____21/11; 3a: 7/6V__G1__0.5/1__0.2/0.25/0___25/9; 3b: 7/6V__G1__0.5/1__0.2/0.25/0___25/9_A; 3c: 7/6V__G1__0.5/1__0.2/0.25/0.5__25/9; 3d: 7/6V__G1__0.5/1__0.2/0.25/0.5_25/9_A; 4a: 3/6V__G1__2/1____0.25/0/0____25/9; 4b: 3/6V__G1__2/1____0.25/0/0____25/9_S; 4c: 3/6V__G1__2/1____0.25/0/0____25/9V; 4d: 3/6V__G1__2/1____0.25/0/0____25/9V_S; 5a: 3/8____G6__2/1____0.25/0/0____25/9; 5b: 3/8____G6__2/1____0.25/0/0____25/9_A; 5c: 3/8____G6__2/1____0.25/0/0____25/9_S; 5d: 3/8____G6__2/1____0.25/0/0____25/9_SA; 6: 8/10___G12_2/1____0.25/0/0____25/9; Different letters above the bars indicate statistically significant differences between variants; n = 6.

Water-holding capacity (WHC) represents the ability of meat to retain water within its structure and is a critical quality parameter of dry-cured ham. Although curing aims to reduce moisture content, it may simultaneously decrease water-binding potential. As shown in Figure 4, WHC ranged from 7.9 to 9.5 g/g of ham, where higher values indicate greater water leakage, and was negatively correlated with mass loss during processing. The correlation coefficient (−0.66) suggests that the free water was removed by the end of curing, while the remaining water was more strongly bound (lower WHC). Despite overall water loss potentially impairing WHC, appropriate protein hydrolysis and pH regulation can mitigate this effect and preserve desirable WHC [46].

The pH of meat, particularly its proximity to the isoelectric point (5.0–5.2), strongly influences protein solubility and water retention, with higher pH enhancing both. Myofibrillar proteins and collagen are the primary contributors to WHC, with myofibrillar proteins responsible for most water retention in muscle tissue. Collagen’s triple-helix structure is stabilized by hydrogen bonds; an increase in pH promotes electrostatic repulsion between helices, allowing the collagen matrix to swell and improving water retention. Conversely, oxidative processes increase protein cross-linking via hydrogen bonds, reducing WHC, which underscores the importance of antioxidants in maintaining hydration. Recent studies have elucidated catechin’s antioxidant mechanism on collagen. Catechin’s molecular size enables penetration into collagen fibrils, stabilizing the structure through inter- and intramolecular hydrogen bonds. These interactions occur primarily between catechin’s B-ring and proline or hydroxyproline residues of collagen [47]. Acerola, rich in phenolic compounds including catechin [48], demonstrated a beneficial effect on reducing water leakage during chamber curing. Nevertheless, even with 2% acerola extract under vacuum conditions, the highest water leakage (high WHC) was recorded. Under vacuum, water distribution across the muscle was more uniform, but less strongly bound water could be more easily expelled.

### 3.5. Shear Force Measurement

**Figure 5:**
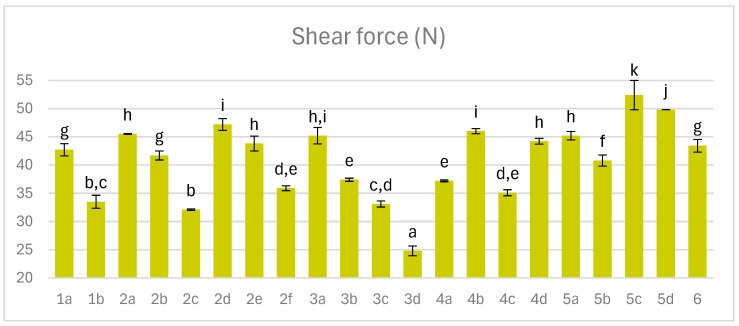
The effect of production technology variants and additives on shear force of dry-cured pork hams; Variants labels (for details see Table 1): 1a: 3/10__G6___0/0___0.2/0/0______17/12_S; 1b: 3/10__G6___0/0___0.2/0/0.5____17/12; 2a: 3/8___G6___0.3/1__0.2/0/0_____21/11; 2b: 3/8___G6___0.3/1__0.2/0/0_____21/11_S; 2c: 3/8___G6___0.3/1__0.2/0/0.5____21/11; 2d: 3/8___G12__0.3/1__0.2/0/0_____21/11; 2e: 3/8___G12__0.3/1__0.2/0/0_____21/11_S; 2f: 3/8___G12__0.3/1__0.2/0/0.5____21/11; 3a: 7/6V__G1__0.5/1__0.2/0.25/0___25/9; 3b: 7/6V__G1__0.5/1__0.2/0.25/0___25/9_A; 3c: 7/6V__G1__0.5/1__0.2/0.25/0.5__25/9; 3d: 7/6V__G1__0.5/1__0.2/0.25/0.5_25/9_A; 4a: 3/6V__G1__2/1____0.25/0/0____25/9; 4b: 3/6V__G1__2/1____0.25/0/0____25/9_S; 4c: 3/6V__G1__2/1____0.25/0/0____25/9V; 4d: 3/6V__G1__2/1____0.25/0/0____25/9V_S; 5a: 3/8____G6__2/1____0.25/0/0____25/9; 5b: 3/8____G6__2/1____0.25/0/0____25/9_A; 5c: 3/8____G6__2/1____0.25/0/0____25/9_S; 5d: 3/8____G6__2/1____0.25/0/0____25/9_SA; 6: 8/10___G12_2/1____0.25/0/0____25/9; Different letters above the bars indicate statistically significant differences between variants; n = 6.

Shear force is a critical indicator of ham tenderness, where lower values denote superior texture and consumer acceptability [49]. Among fermented dry-cured hams, shear force exhibited the greatest variability, ranging from 24.8 to 52.4 N (Figure 5). This parameter is influenced by proteolysis, water content, and water-binding capacity. Typically, shear force correlates negatively with moisture, increasing as water decreases. However, in this study, the correlation between shear force and mass loss was +0.13, suggesting that other factors—such as environmental conditions and ingredient composition—played a more decisive role in shaping texture.

A marked reduction in shear force was observed in hams inoculated with mold (variants 1b, 2c,f, 3c,d), attributable to the proteolytic activity of *P. nalgiovense*, which accelerates protein degradation. Additional determinants include curing time, temperature, and endogenous enzyme activity, all of which influence muscle structure and tenderness. Cathepsin activity is suppressed at pH above 5.40, indicating that aging at 25 °C with relatively low pH should favor protease function and enhance ham tenderness. However, during curing in the chamber, the inoculation with mold was more important for the tenderization of the meat. Nevertheless, excessive proteolysis may lead to undesirable pastiness, necessitating control through adequate salt levels [30]. The 3d variant showed such soft consistency, below 30 N.

### 3.6. Lipid Oxidation Index (TBARS)

Dry-cured ham lipids comprise triacylglycerols and membrane lipids, with free fatty acids—highly susceptible to oxidation—reaching up to 20% by the end of processing. The lipid profile typically includes 35–40% saturated, 45–50% monounsaturated, and 10–15% polyunsaturated fatty acids, while oxidation products such as aldehydes fluctuate throughout curing [50]. Lipid oxidation is commonly assessed using thiobarbituric acid reactive substances (TBARS), where higher TBARS values indicate greater oxidative degradation.

**Figure 6:**
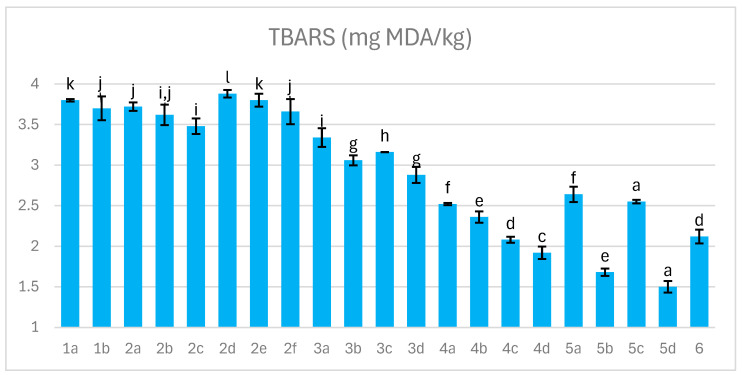
The effect of production technology variants and additives on TBARS values of dry-cured pork hams; Variants labels (for details see Table 1): 1a: 3/10__G6___0/0___0.2/0/0______17/12_S; 1b: 3/10__G6___0/0___0.2/0/0.5____17/12; 2a: 3/8___G6___0.3/1__0.2/0/0_____21/11; 2b: 3/8___G6___0.3/1__0.2/0/0_____21/11_S; 2c: 3/8___G6___0.3/1__0.2/0/0.5____21/11; 2d: 3/8___G12__0.3/1__0.2/0/0_____21/11; 2e: 3/8___G12__0.3/1__0.2/0/0_____21/11_S; 2f: 3/8___G12__0.3/1__0.2/0/0.5____21/11; 3a: 7/6V__G1__0.5/1__0.2/0.25/0___25/9; 3b: 7/6V__G1__0.5/1__0.2/0.25/0___25/9_A; 3c: 7/6V__G1__0.5/1__0.2/0.25/0.5__25/9; 3d: 7/6V__G1__0.5/1__0.2/0.25/0.5_25/9_A; 4a: 3/6V__G1__2/1____0.25/0/0____25/9; 4b: 3/6V__G1__2/1____0.25/0/0____25/9_S; 4c: 3/6V__G1__2/1____0.25/0/0____25/9V; 4d: 3/6V__G1__2/1____0.25/0/0____25/9V_S; 5a: 3/8____G6__2/1____0.25/0/0____25/9; 5b: 3/8____G6__2/1____0.25/0/0____25/9_A; 5c: 3/8____G6__2/1____0.25/0/0____25/9_S; 5d: 3/8____G6__2/1____0.25/0/0____25/9_SA; 6: 8/10___G12_2/1____0.25/0/0____25/9; Different letters above the bars indicate statistically significant differences between variants; n = 6.

Monitoring TBARS is essential for ensuring product quality and shelf life, as levels exceeding 2 mg MDA/kg generally reduce consumer acceptability. Figure 6 presents TBARS values detected in dry-cured hams. Factors such as salt concentration and curing method influence TBARS levels; NaCl content of 3% or higher may lower TBARS values [51]. In this study, TBARS ranged from 1.5 to 3.9 mg MDA/kg, indicating that salt alone was insufficient to maintain oxidative stability. Significant reductions were achieved through antioxidants such as acerola and ginger extracts, particularly in variants 4–6. Vacuum curing, by limiting oxygen exposure, further reduced oxidative spoilage and preserved lipid and protein integrity, and the use of an additional stocking cover also contributed positively lowering TBARS (variants 4c,d, 5b,d) [52].

Comparable TBARS levels in trial 6 may be attributed to high antioxidant addition combined with advanced lactic acid fermentation, supported by elevated glucose supplementation. This might promote the formation of bioactive peptides, antioxidant enzymes, and chelation of pro-oxidant metal ions by lactic acid bacteria (LAB). The acidic environment created during fermentation further inhibited lipid oxidation, enhancing oxidative stability. Higher TBARS values were observed in batches inoculated solely with *L. sakei*, whereas combined starter cultures yielded lower TBARS levels. Variants 1–4a,b exhibited markedly high TBARS values due to insufficient radical protection resulting from generally low nitrite concentration and limited synergistic effects of additives and processing conditions applied in these variants [52]. Smoking, although less pronounced, contributed to TBARS reduction, likely due to smoke-derived compounds with antioxidant properties [43,53].

### 3.7. Color in the CIEL*a*b* System

**Figure 7:**
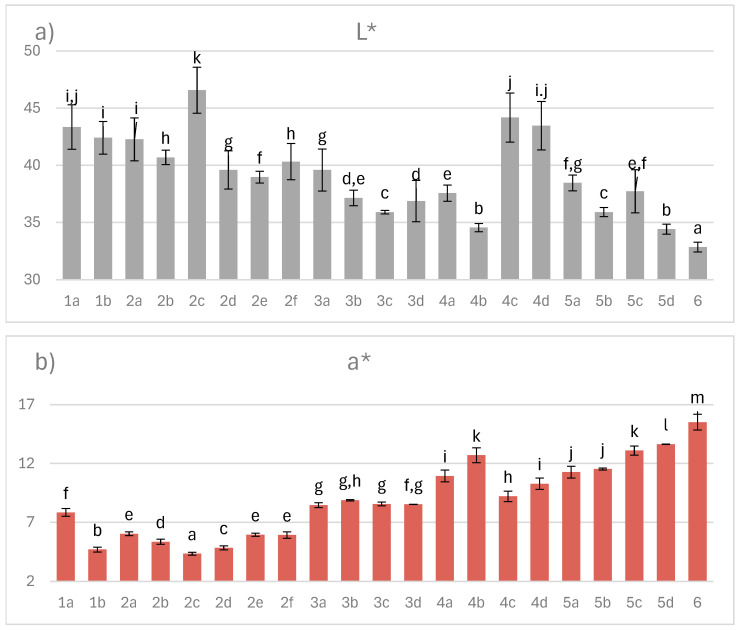

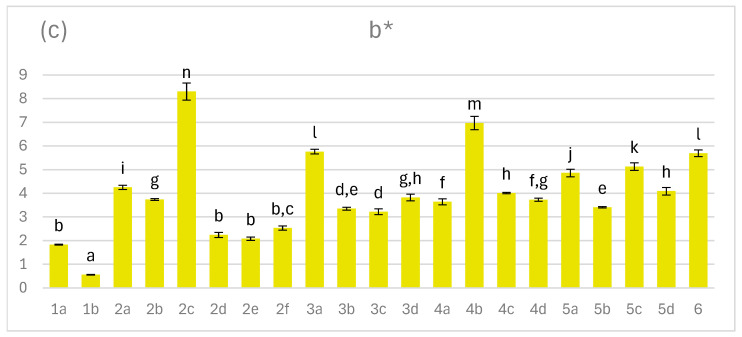
The effect of production technology variants and additives on color parameters of dry-cured pork hams: (**a**) L* lightness, (**b**) a* red, (**c**) b* yellow; Variants labels (for details see Table 1): 1a: 3/10__G6___0/0___0.2/0/0______17/12_S; 1b: 3/10__G6___0/0___0.2/0/0.5____17/12; 2a: 3/8___G6___0.3/1__0.2/0/0_____21/11; 2b: 3/8___G6___0.3/1__0.2/0/0_____21/11_S; 2c: 3/8___G6___0.3/1__0.2/0/0.5____21/11; 2d: 3/8___G12__0.3/1__0.2/0/0_____21/11; 2e: 3/8___G12__0.3/1__0.2/0/0_____21/11_S; 2f: 3/8___G12__0.3/1__0.2/0/0.5____21/11; 3a: 7/6V__G1__0.5/1__0.2/0.25/0___25/9; 3b: 7/6V__G1__0.5/1__0.2/0.25/0___25/9_A; 3c: 7/6V__G1__0.5/1__0.2/0.25/0.5__25/9; 3d: 7/6V__G1__0.5/1__0.2/0.25/0.5_25/9_A; 4a: 3/6V__G1__2/1____0.25/0/0____25/9; 4b: 3/6V__G1__2/1____0.25/0/0____25/9_S; 4c: 3/6V__G1__2/1____0.25/0/0____25/9V; 4d: 3/6V__G1__2/1____0.25/0/0____25/9V_S; 5a: 3/8____G6__2/1____0.25/0/0____25/9; 5b: 3/8____G6__2/1____0.25/0/0____25/9_A; 5c: 3/8____G6__2/1____0.25/0/0____25/9_S; 5d: 3/8____G6__2/1____0.25/0/0____25/9_SA; 6: 8/10___G12_2/1____0.25/0/0____25/9; Different letters above the bars indicate statistically significant differences between variants; n = 6.

In addition to juiciness, tenderness, and flavor, color remains a critical quality attribute of cured meats, strongly influencing consumer purchasing decisions. The characteristic pink-red hue of cured ham originates from the interaction between myoglobin and nitric oxide, whereas deviations toward pale, yellow, or greenish tones indicate undesirable oxidative changes. As illustrated in Figure 7, the L* values of dry-cured ham ranged from 32.9 to 46.6, a* from 4.3 to 15.5, and b* from 0.56 to 8.30, confirming that multiple processing parameters exert a significant impact on color development.

Although color is primarily a visual trait, consumers often subconsciously associate it with the expected flavor profile of dry-cured meat [43]. The pH level also modulates color expression; lower pH values tend to produce lighter shades due to increased water release and enhanced light reflection by myoglobin. Notably, the incorporation of acerola extract—naturally rich in red pigments—proved decisive, reinforcing the red hue even under conditions of reduced nitrite content. Conversely, the emergence of yellow tones was linked to several factors, including the addition of ginger extract.

### 3.8. Nitrite and Nitrate Content

**Figure 8:**
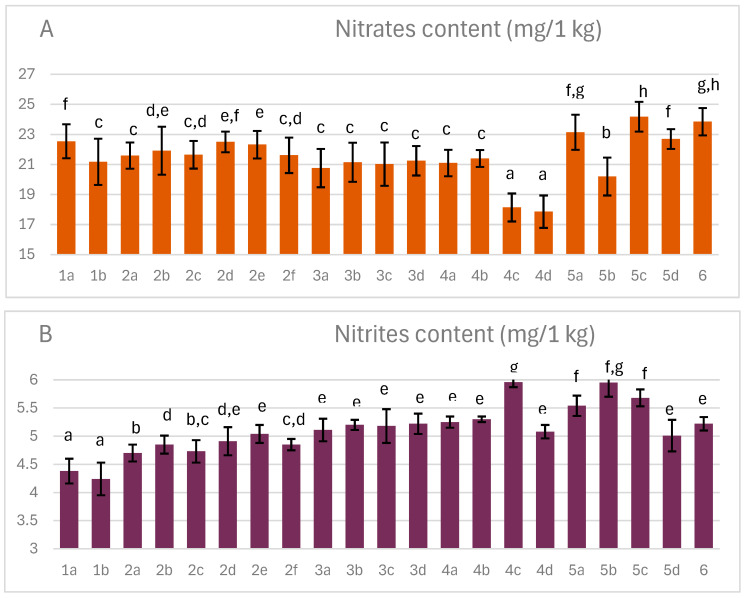
The effect of production technology variants and additives on nitrates and nitrites content of dry-cured pork hams: (**A**) nitrates; (**B**) nitrites; Variants labels (for details see Table 1): 1a: 3/10__G6___0/0___0.2/0/0______17/12_S; 1b: 3/10__G6___0/0___0.2/0/0.5____17/12; 2a: 3/8___G6___0.3/1__0.2/0/0_____21/11; 2b: 3/8___G6___0.3/1__0.2/0/0_____21/11_S; 2c: 3/8___G6___0.3/1__0.2/0/0.5____21/11; 2d: 3/8___G12__0.3/1__0.2/0/0_____21/11; 2e: 3/8___G12__0.3/1__0.2/0/0_____21/11_S; 2f: 3/8___G12__0.3/1__0.2/0/0.5____21/11; 3a: 7/6V__G1__0.5/1__0.2/0.25/0___25/9; 3b: 7/6V__G1__0.5/1__0.2/0.25/0___25/9_A; 3c: 7/6V__G1__0.5/1__0.2/0.25/0.5__25/9; 3d: 7/6V__G1__0.5/1__0.2/0.25/0.5_25/9_A; 4a: 3/6V__G1__2/1____0.25/0/0____25/9; 4b: 3/6V__G1__2/1____0.25/0/0____25/9_S; 4c: 3/6V__G1__2/1____0.25/0/0____25/9V; 4d: 3/6V__G1__2/1____0.25/0/0____25/9V_S; 5a: 3/8____G6__2/1____0.25/0/0____25/9; 5b: 3/8____G6__2/1____0.25/0/0____25/9_A; 5c: 3/8____G6__2/1____0.25/0/0____25/9_S; 5d: 3/8____G6__2/1____0.25/0/0____25/9_SA; 6: 8/10___G12_2/1____0.25/0/0____25/9; Different letters above the bars indicate statistically significant differences between variants; n = 6.

The residual nitrate and nitrite content in the finished product was significantly lower than that initially added to the ham before aging and curing, averaging approximately 25 mg/kg (Figure 8), representing a more than three-fold reduction from 80 mg/kg, in line with regulations [7].

Residual nitrate and nitrite levels varied between the variants, primarily due to the drip loss of these compounds along with the juices during aging and curing. Furthermore, the conversion of nitrites to nitric oxide under the influence of antioxidant activity is known [54]. These were present in the added acerola and ginger extracts, particularly the ascorbic acid supplied with acerola. However, despite these changes, nitrites were still present in the mature ham. This is due to the fact that the conversion of nitrites to nitric oxide, which reacts with hemoglobin, does not involve all available nitrites. Furthermore, some nitrites can be formed as a result of the reduction of nitrates to nitrites by certain microorganisms, including lactic acid bacteria [55].

With regard to that, the lowest nitrate content was observed in variants 4c and d, where vacuum maturation was used and weight losses were the lowest. The highest nitrate content was recorded in variants 1a, 5a, c, d, and 6, which were characterized by high weight loss. However, other factors may also have played a role, as the correlation between these two parameters was 0.83. These factors may include smoking, which in the initial phase causes significant losses of soluble substances along with the released juices. Smoking may also contribute to the formation of nitrosamines with nitrites. In the case of nitrite, the highest levels were observed in variants 1a and b, in which no plant extracts were added. However, the presence of antioxidants from acerola and ginger, as well as the increased addition of bacterial starters, favored higher nitrite concentrations. The highest residues were observed in variants 4c and 5b, in which juice flow was additionally limited by the use of vacuum ripening or an additional stocking.

### 3.9. Overall Acceptability

**Figure 9:**
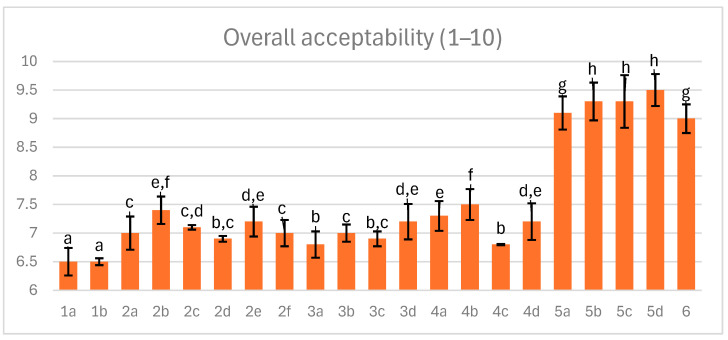
The effect of production technology variants and additives on overall acceptability of dry-aged pork hams; Variants labels (for details see Table 1): 1a: 3/10__G6___0/0___0.2/0/0______17/12_S; 1b: 3/10__G6___0/0___0.2/0/0.5____17/12; 2a: 3/8___G6___0.3/1__0.2/0/0_____21/11; 2b: 3/8___G6___0.3/1__0.2/0/0_____21/11_S; 2c: 3/8___G6___0.3/1__0.2/0/0.5____21/11; 2d: 3/8___G12__0.3/1__0.2/0/0_____21/11; 2e: 3/8___G12__0.3/1__0.2/0/0_____21/11_S; 2f: 3/8___G12__0.3/1__0.2/0/0.5____21/11; 3a: 7/6V__G1__0.5/1__0.2/0.25/0___25/9; 3b: 7/6V__G1__0.5/1__0.2/0.25/0___25/9_A; 3c: 7/6V__G1__0.5/1__0.2/0.25/0.5__25/9; 3d: 7/6V__G1__0.5/1__0.2/0.25/0.5_25/9_A; 4a: 3/6V__G1__2/1____0.25/0/0____25/9; 4b: 3/6V__G1__2/1____0.25/0/0____25/9_S; 4c: 3/6V__G1__2/1____0.25/0/0____25/9V; 4d: 3/6V__G1__2/1____0.25/0/0____25/9V_S; 5a: 3/8____G6__2/1____0.25/0/0____25/9; 5b: 3/8____G6__2/1____0.25/0/0____25/9_A; 5c: 3/8____G6__2/1____0.25/0/0____25/9_S; 5d: 3/8____G6__2/1____0.25/0/0____25/9_SA; 6: 8/10___G12_2/1____0.25/0/0____25/9; Different letters above the bars indicate statistically significant differences between variants; n = 6.

Dry-cured meat acquires a distinctive flavor and aroma through proteolysis and lipolysis. The resulting compounds are relatively concentrated due to the loss of significant amounts of water during the process. Dry-curing imparts aromas of roast, butter, nuts, toast, and sweets to the meat, while the high glutamate content in pork gives processed meats an umami flavor. Dry curing also improves the tenderness and juiciness of pork. As is presented in Figure 9 the overall acceptability values ranged from 6.5 to 9.5. Overall acceptability is a combination of sensory attributes such as texture, tenderness, off-flavor, flavor, saltiness and sourness. They depend on many factors, among which salt content is significant. Sodium chloride is a strong inhibitor of most proteases involved in ham curing. Hams with reduced salt content (about 3% added to raw meat) generally showed higher sensory scores. Therefore, this salt level was used in the study.

Excessive protease activity can lead to deep proteolysis, causing textural defects like pastiness and excessive softness, as well as intensified flavors such as saltiness and bitterness. The lack of excessive off-flavors like bitterness in deeply fermented samples (variants 5–6; higher curing temperature at high glucose level) suggests that proteolysis was well-controlled, avoiding the accumulation of undesirable free amino acids. Maintaining optimal pH (not negatively affecting flavor) and salt content was essential for high sensory evaluation in these variants, with a shear force around 45 N considered acceptable [56,57,58].

The best-rated hams had a relatively low pH of around 5.3, with a moderate glucose addition 6 g/kg, cured at 25 °C, and included one bacterial starter culture. The correlation between lower pH and improved sensory scores supports previous findings that moderate acidification enhances flavor development and microbial safety.

The hams were microbiologically safe, as demonstrated by routine microbiological purity testing. According to the result of the reference methods (EN ISO 6888-1:2022-03/A1:2024-02, EN ISO 16649-2:2004; EN ISO 11290-1:2017-07; EN ISO 6579-1:2017-04.A1:2020-09; EN ISO 4833-1:2013, respectively), the number of coagulase-positive *Staphylococci* and beta-glucuronidase-positive *Escherichia* coli was <10 cfu/g, *Listeria monocytogenes* and *Salmonella* spp. were not detected, and the total number of microorganisms was 1.2 × 10^6^ cfu/g. These hams showed relatively low lipid oxidation levels and minimal expressible moisture. They had high lightness, high red pigment levels, and moderately high yellow pigment levels.

**Figure 10:**
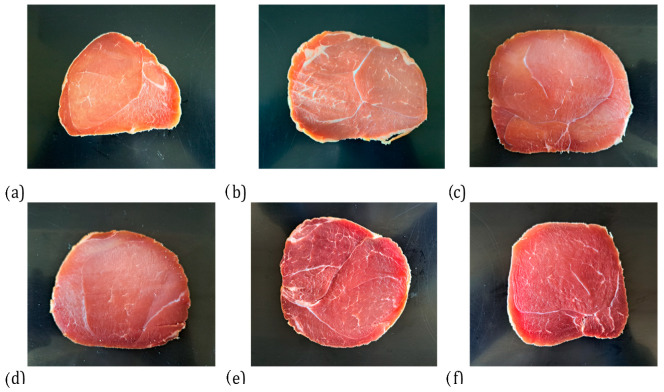
A cross-sectional view of hams dry-cured in the presence of *L. sakei*, with increasing con-tent of acerola extract; according to Table 1: (**a**) variant 1b; (**b**) variant 2a; (**c**) variant 3a; (**d**) variant 4a; (**e**) variant 5a; (**f**) variant 6. Variants labels (for details see Table 1): 1b: 3/10__G6___0/0___0.2/0/0_____17/12; 2a: 3/8___G6___0.3/1__0.2/0/0_____21/11; 3a: 7/6V__G1__0.5/1__0.2/0.25/0___25/9; 4a: 3/6V__G1__2/1____0.25/0/0____25/9; 5a: 3/8____G6__2/1____0.25/0/0___25/9; 6: 8/10___G12_2/1____0.25/0/0___25/9; Different letters above the bars indicate statistically significant differences between variants; n = 6.

Figure 10 shows a cross-sectional view of hams dry-cured in the presence of *L. sakei*, with increasing content of acerola extract which highlights the bright red color of the meat, which increases the attractiveness of the ham. Due to proteolytic and lipolytic activity, the presence of mold contributed to the characteristic flavor and texture of dry-cured ham through the production of various metabolites during fermentation. Mold on the surface improved sensory quality by providing a juicier texture and reducing the risk of curing defects such as surface drying and cracking [59,60]. However, the presence of mold itself is not particularly attractive to consumers, and hams with mold starter cultures did not receive high acceptability scores in the study. On the other hand, phenols penetrating the ham during smoking are positively correlated with smoky aroma. Due to the distinct volatile profile and pronounced smoky flavor, smoked ham can be distinguished from other types of dry-cured hams.

The study used curing temperatures ranging from 17 to 25 °C. Dry-curing at 25 °C resulted in lower shear force due to higher proteolysis, and thus better consumer acceptance compared to lower-temperature curing, especially when plant extracts from acerola and ginger were added. These plant extracts reduced lipid oxidation levels, keeping oxidation products within acceptable limits, which could otherwise be elevated due to the presence of divalent metal cations from sea salt [61]. The combination of acerola and ginger extracts not only reduced lipid oxidation but also supported proteolysis, contributing to improved tenderness and flavor complexity [16]. However, it should be borne in mind that adding acerola to food products, including meat, can pose potential health risks, particularly for individuals with latex allergies. Acerola contains proteins that exhibit strong cross-reactivity with latex allergens, which means that even trace amounts can trigger severe allergic reactions, including urticaria, swelling of the lips, respiratory distress, and in extreme cases, anaphylaxis [62]. Ginger is generally recognized as safe (GRAS) in normal food amounts, with mild, temporary side effects typically occurring at high supplement doses, affecting the digestive tract, and rarely interacting with medications [63].

The study revealed a discrepancy between what consumers focus on when making choices—expected acceptability—and what they actually experience. This suggests that visual preferences for lighter-colored hams, not covered with mold, may prevent them from choosing ham cured with mold, which could satisfy their sensory preferences for taste and texture, and additionally give health benefits. The latter, however, may only become apparent after regular and long-term consumption [64,65,66,67]. It highlights that current knowledge about the quality of fermented, dry-cured hams allows for more optimized production and the development of characteristics, such as color and surface quality. However, the use of these practices should be accompanied by consumer education on the correlations between various ham quality characteristics.

## 4. Conclusions

New EU regulations coming into force at the end of 2025 require lower nitrite concentrations in ham, including dry-cured hams. The study showed that sensory acceptability with such reduced nitrite levels can be achieved by enriching the meat with plant extracts that enhance red color, act as antioxidants, and lower pH. The results demonstrated that the synergistic use of natural plant extracts (acerola and ginger) and starter cultures of bacteria and mold enables the production of high-quality and safe dry-cured ham. Acerola extract rich in antioxidants played a crucial role in stabilizing the red color of the meat by enhancing visual appeal through its natural red hue. It also contributed to lowering TBARS values to below 2 mg MDA/kg, indicating reduced lipid oxidation. Ginger extract, with its antioxidant properties and proteolytic enzymes, supported yellow pigment development (increased b* values), improved tenderness noticed as reduced shear force, enhancing juiciness and overall sensory quality.

Both extracts significantly reduced lipid oxidation, especially when used in combination with microbial starter cultures and vacuum conditions during curing (120 mBa), which also limited mass loss to 22–24%, improving juiciness, although not directly linked to protein water-holding capacity. The use of *Lactobacillus sakei* and *Pediococcus acidilactici* starter cultures accelerated fermentation, reduced pH to optimal levels (5.6–5.7), and maintained microbial safety. The natural acidity of acerola further supported pH reduction and proteolysis. Inoculation with *Penicillium nalgiovense* facilitated ham softening, contributing to desirable texture. The addition of 0.6% glucose was identified as the most beneficial, serving as an effective carbon source for bacterial starters. Smoking further enhanced sensory characteristics and contributed to reduced lipid oxidation. Samples containing both acerola and ginger extracts, fermented at 25 °C with bacterial starter, achieved the highest overall acceptability scores (up to 9.0/9.5). These variants were characterized by balanced flavor, tenderness, and color, aligning well with consumer preferences.

## 5. Study Limitations

This study did not include analysis of specific microbiological changes in the meat or the degree of proteolysis. We intend to investigate this further, along with the content and identification of bioactive peptides and biogenic amines, as well as the specific characteristics available by using e-nose and e-tongue, as this is of interest both in terms of food safety and potential health benefits. Future research will include water activity measurements to further validate the relationship between fermentation intensity and product stability.

## Figures and Tables

**Table 1:** Variants of dry curing ham differing in process conditions and additives used.

Pork Sample	1		2						3				4				5				6
Ingredient/Stag	1a	1b	2a	2b	2c	2d	2e	2f	3a	3b	3c	3d	4a	4b	4c	4d	5a	5b	5c	5d	
tend erizing	3 °C RH 80% 3 days																				
Tumbling with seasonings a ging with sea sonings	3 °C 4 h		3 °C 4 h						3 °C 4 h (first 3 h without starter)				3 °C 4 h				3 °C 4 h				3 °C 4 h
	3 °C 10 days		3 °C 8 days						7 °C 6 days in vacuum 800 mBa				3 °C 6 days in vacuum 900 mBa				3 °C 8 days				8 °C 10 days
	Seasonings g/kg *																				
Sea salt	10																				
Mineral salt	19.92																				
Nitrite	0.08																				
Glucoses	6		6	6	6	12	12	12	1					1				6			12
*Lactobacillus sakei*	0.2				0.2					0.2				0.25				0.25			0.25
Acerola	-				0.3					0.5				2				2			2
Ginger	-				1					1				1				1			1
*Pediococcus acidilactici*	-				-					0.25				-				-			-
curing	17 °C 12 days			21 °C 11 days					25 °C 9 days				25 °C 9 days				25 °C 9 days				25 °C 9 days
Vacuum curing 120 mBa	-		-						-				-		+			-			-
Additional stocking	-				-				-	+	-	+		-		-		+	-	+	-
*Penicillium nalgiovense*	-	0.5	-	-	0.5	-	-	0.5	-	-	0.5	0.5		-					-		-
Smoking	3 h 20 °C	-	-	3 h 20 °C	-	-	3 h 20 °C	-			-		-	3 h 20 °C	-	3 h 20 °C	-	-	3 h 20 °C	3 h 20 °C	-
Stabilization	14 °C 6 days																				

* The amount of all additives is expressed in g/ kg, in the case of microbial starters it refers to the mass of the preparation on the carrier.

## Data Availability

The original contributions presented in the study are included in the article. Further inquiries can be directed to the corresponding author.
